# Predicting autism spectrum disorder severity in children based on specific language milestones: a random forest model approach

**DOI:** 10.1186/s13034-025-00988-0

**Published:** 2025-11-18

**Authors:** Haiyi Xiong, Xueli Xiang, Xiao Liu, Ting Yang, Jinjin Chen, Jie Chen, Tingyu Li

**Affiliations:** 1 Growth, Development and Mental Health Center of Children and Adolescents, Chongqing Key Laboratory of Child Neurodevelopment and Cognitive Disorders, Ministry of Education Key Laboratory of Child Development and Disorders, National Clinical Research Center for Child Health and Disorders, Children’s Hospital of Chongqing Medical University, Chongqing, China; 2 Department of Pediatrics, Department of Pediatrics, NHC Key Laboratory of Birth Defects and Reproductive Health, Chongqing Health Center for Women and Children, Women and Children’s Hospital of Chongqing Medical University, Chongqing, China; 3 Department of Child Healthcare, Shanghai Children’s Hospital, Shanghai Jiao Tong University, Shanghai, China

**Keywords:** Autism, Language development, Random forest, Predictive model

## Abstract

**Background:**

Language impairments are among the most prevalent co-occurring conditions in children with autism spectrum disorder (ASD), and delayed language milestones often serve as early developmental warning signs. However, it remains unclear whether specific language milestones can reliably predict the severity of ASD symptoms, particularly in regions where there is a long delay between initial screening and formal diagnosis.

**Methods:**

This study included 574 children diagnosed with ASD, stratified into two age groups: under 4 years (*n* = 288) and 4 years or above (*n* = 286). A total of 33 language milestone items covering receptive, expressive, and pragmatic aspects were evaluated. The Boruta algorithm was applied to identify significant predictors of symptom severity, and random forest models were constructed separately for each age group. Nested cross-validation and grid search were used for hyperparameter tuning. Model performance was assessed using bootstrapping with 1,000 replications to estimate area under the receiver operating characteristic curve (AUC), accuracy, sensitivity, specificity, and F1 scores.

**Results:**

In children under 4 years, 14 features were identified as significant predictors of ASD severity, with “Identifies 1 picture” and “Expresses demands by language” ranked highest. In children aged 4 years and above, 16 features were significant, with “Identifies 2 colors” and “Calls partner by name” being the most influential. The random forest models demonstrated robust predictive performance, with AUC values of 0.81 ± 0.01 (younger group) and 0.85 ± 0.00 (older group).

**Conclusion:**

Our findings suggest that specific early language milestones, particularly those reflecting pragmatic abilities, may serve as valuable predictors of ASD severity. Leveraging these milestones in clinical practice could support earlier severity stratification and facilitate more tailored intervention planning, particularly in primary care settings.

**Supplementary Information:**

The online version contains supplementary material available at 10.1186/s13034-025-00988-0.

## Introduction

Autism spectrum disorder (Autism spectrum disorders, ASD) is a complex neurodevelopmental condition defined by two core symptom domains: (1) persistent deficits in social communication and social interaction across multiple contexts, and (2) restricted, repetitive patterns of behavior, interests, or activities [1]. Language impairment is one of the most common comorbidities in children with ASD, with approximately 87% of 3-year-old children with ASD exhibiting delayed language development [2]. Language skills are closely associated with cognitive functioning and adaptive behavior in individuals with ASD, and are considered one of the most important predictors of long-term outcomes [3, 4].

### Early language development as a predictor of severity in ASD

While the core symptom domains define the condition, its clinical severity is typically quantified using standardized assessment tools such as the Childhood Autism Rating Scale (CARS), which is widely used in clinical practice [5]. Language ability plays a crucial role in the overall development of children with ASD, particularly in the domain of social communication [6]. As observable and quantifiable indicators in early childhood, language milestones offer valuable clinical utility. Specifically, the assessment of language milestones in children with ASD—such as the age at which the first word or first phrase is spoken—is one of the most frequently reported indicators for predicting later language outcomes [7–9]. If ASD severity can be estimated from early language development, it may provide an additional reference for individualized intervention planning, particularly in settings where access to comprehensive assessments is limited. However, the predictive value of language milestones for symptom severity in children with ASD remains a subject of debate. Some studies have shown that structural language impairments in childhood predict later adaptive and behavioral outcomes [10], whereas others reported that early language abilities are more strongly related to communication skills than to autism symptoms [9, 11]. In contrast, findings also indicate that the age at first phrase is significantly associated with developmental level and adaptive functioning, particularly in more severe subgroups [12]. It is worth noting that most of the aforementioned studies exploring language-related predictors of ASD symptoms have largely focused on early vocabulary and morphosyntactic development, such as the age at first word or first phrase production, or on overall language developmental delays. Therefore, we hypothesize that among the atypical sequence of language milestone acquisition in children with ASD, there may exist specific predictors with stronger predictive power for ASD severity—especially as quantified by CARS thresholds.

In our previous research, we employed a nonparametric item response theory model—the Mokken scale analysis—to quantitatively describe both the overall pattern and developmental sequence of language milestones in children with ASD [13]. This assessment encompassed not only receptive and expressive language domains, but also a series of items related to pragmatic language use. Building on this comprehensive evaluation, the present study aims to predict ASD severity in children by applying a machine-learning model to the acquisition of specific early language milestones.

### Machine learning algorithm models

Machine learning models have gained significant traction across a range of disciplines due to their capacity to model complex, nonlinear relationships in data—offering notable advantages over traditional statistical methods. These models have shown considerable promise in areas such as disease diagnosis, subtype classification, and biomarker discovery [14–16].

In recent years, many researchers have developed machine learning-based predictive models for the screening and diagnosis of ASD [17]. Yuwattana et al. demonstrated that using a random forest algorithm on 27 selected items from the Autism Diagnostic Interview-Revised (ADI-R) could effectively distinguish children with ASD from non-ASD controls, achieving a sensitivity of 99.24% and a specificity of 61.35% [18]. However, despite being one of the gold standards for ASD diagnosis, ADI-R data are not widely accessible at the primary care level and are often difficult to obtain in a timely manner. Other researchers have explored the use of electronic medical records (EMRs) for ASD screening [19, 20]. For example, Dick et al. employed a Transformer-based ensemble model that integrated maternal (prenatal) and offspring (fetal and postnatal) characteristics, achieving an area under the receiver operating characteristic curve (AUC) of 69.6%, with a sensitivity of 70.9% and a specificity of 56.9% for predicting ASD diagnosis [21]. These findings suggest that predictive models relying solely on clinical data—often constrained by subjective reporting, heterogeneous data quality, and the lack of objective biomarkers—may have limitations in diagnostic accuracy compared with approaches that integrate multimodal or biological measures. Further studies have attempted to predict ASD using data derived from facial expression analysis [22], neuroimaging [23], eye-tracking [24], and even genetic information [25]. While promising, these approaches rely on specialized technologies and equipment, which may limit their feasibility and scalability in routine clinical practice.

In summary, this study aims to predict ASD severity in children by applying a machine-learning model to the acquisition of specific early language milestones, with ASD severity classified according to CARS thresholds. Utilizing easily observable language milestones for rapid severity assessment is especially valuable in regions where comprehensive standardized tools for evaluating ASD severity are not readily available or widely implemented. This approach has the potential to support earlier stratification and more individualized intervention planning among children with a confirmed ASD diagnosis, ultimately contributing to improved long-term outcomes.

## Methods

### Study participants

**Fig. 1:**
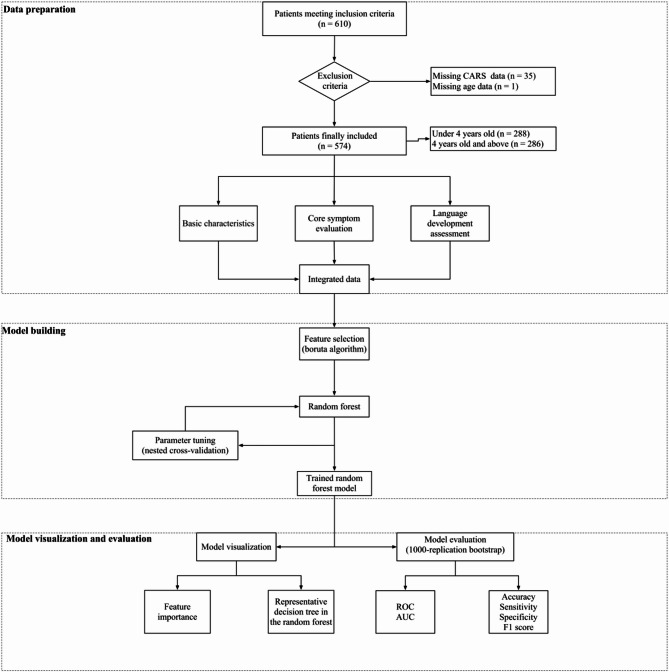
The framework of this study

The framework of this study is illustrated in Fig. 1. This study was designed as a cross-sectional investigation. The data source is consistent with our previously published study [13] and includes 610 children aged 2–7 years with a confirmed diagnosis of ASD, all of whom had complete language milestone data. The ASD diagnosis was confirmed by a developmental pediatrician and psychologist at the Children’s Hospital of Chongqing Medical University and the Children’s Hospital of Shanghai according to the Diagnostic and Statistical Manual of Mental Disorders, Fifth Edition (DSM-5) criteria. Participants were consecutively recruited from the Chongqing and Shanghai regions between 2018 and 2022, all of whom were raised in a monolingual Chinese-speaking environment. All study variables were defined a priori and collected simultaneously during standardized clinical assessments at the time of each child’s inclusion. The study received approval from the Ethics Committee of the Children’s Hospital of Chongqing Medical University [Approval No: (2018) IRB (STUDY) NO.121]. Additionally, this study is registered with the China Clinical Trial Registry (Registration Number: ChiCTR2000031194). Written informed consent was obtained from the caregivers of all participants.

The exclusion criteria were as follows: (1) a history of other neurological or genetic diseases, such as epilepsy, cerebral palsy, Rett’s syndrome, or severe head injury, (2) significant sensory or motor impairment caused by organic disease, (3) missing data, including missing CARS scores or age data.

### Variables selection

Our study meticulously selected variables encompassing (1) basic information (chronological age, sex), (2) neurodevelopmental assessment conducted by the Chinese-adapted version of the Gesell Developmental Scale (GDS), and (3) core symptom evaluation (ASD severity assessed by CARS).

The acquisition of language milestones was extracted from the GDS, from which we extracted 33 previously identified ASD specific language milestone items, including 18 expressive language (EL) and 15 receptive language (RL) items. The items and their corresponding theoretical acquisition ages are presented in Table S1.

Notably, the independent variable was defined as the difference between the theoretical acquisition and the actual acquisition for each language milestone. The theoretical ages of acquisition for each language milestone were derived from the GDS, which delineates the expected developmental timeline for typically developing children to attain each skill. For each child with ASD, milestones with a theoretical age at or below the child’s chronological age were coded as theoretically acquired (1), and those above were coded as not yet theoretically acquired (0). Actual acquisition of each milestone was recorded during the GDS assessment as 1 (acquired) or 0 (not acquired). We then calculated the absolute difference between the theoretical and actual status for each milestone. A value of 0 indicated that the child’s acquisition matched the theoretical expectation, whereas a value of 1 indicated a deviation—either a milestone expected but not acquired, or acquired earlier than expected. These binary deviation indicators were used as independent variables in the subsequent analyses. Given that language development in ASD can deviate in both delays and occasional earlier-than-expected acquisition, all milestones were initially included in the feature set to capture this variability.

The dependent variable was the severity of ASD in children, assessed using the CARS, which was selected given its broad clinical use, greater feasibility compared with the ADI-R, and routine implementation at our center that ensured standardized and comprehensive data collection. A CARS score >36 indicates severe autism symptoms (class = 1), while a score ≤ 36 indicates less severe symptoms (class = 0) [26].

Although the theoretical sequence of milestones was established for the entire 2-7-year-old cohort using Mokken scale analysis, the predictive utility of individual milestones varies by developmental stage. To avoid ceiling effects in older children and floor effects in younger children, which are well documented in developmental assessments [27], we divided the sample into two subgroups—children under 4 years old and those aged 4 years and above—and construct separate models, allowing the algorithm to capture age-appropriate discriminative features.

### Feature selection and model optimization

To identify the most relevant predictors of ASD severity in children, we applied the Boruta algorithm for feature selection. In this algorithm, “maxRuns” controls the maximum number of iterations to achieve feature stability, and “pValue” defines the significance threshold for determining whether a feature is confirmed or rejected. This method is well-suited for high-dimensional data, as it creates shadow features by randomly permuting the original variables and evaluates feature importance using a random forest classifier. Features that consistently exhibit lower importance than the most informative shadow feature are progressively eliminated, ensuring that only those with meaningful predictive power are retained.

Subsequent to the feature selection process, we constructed the predictive model using the random forest algorithm. To fine-tune the model and mitigate the limitations posed by a relatively small sample size, we adopted a nested cross-validation framework in conjunction with grid search. Rather than dividing the dataset into fixed training and testing subsets, we implemented a 4-fold cross-validation scheme within both the inner and outer loops of the nested design. This technique supports a more reliable assessment of model performance by evaluating various hyperparameter combinations across multiple data partitions [28, 29]. The key hyperparameters optimized included the number of trees (n_estimators) and the maximum tree depth (max_depth), with the final parameter set selected based on the combination that maximized the AUC.

Within the inner loop, grid search was used to systematically investigate different parameter settings. Each configuration was evaluated through 4-fold cross-validation, and the performance results were visualized using heatmaps to facilitate the selection of optimal hyperparameters. This nested approach helps to reduce overfitting and enhances the model’s generalizability by ensuring that parameter tuning is consistently validated across diverse subsets of the data, ultimately improving predictive performance [30].

### Model visualization and performance evaluation

To improve model interpretability, we examined feature importance scores, highlighting the variables that had the greatest impact on prediction outcomes. Additionally, we visualized two representative decision trees from the random forest ensemble to illustrate how individual trees contributed to the aggregated prediction process. This provided valuable insight into the internal decision-making structure of the model.

For performance evaluation, we employed the bootstrap resampling technique with 1,000 iterations to generate robust estimates of key classification metrics, including accuracy, sensitivity, specificity, F1 score, and the AUC. The ROC curve analysis served as a critical tool for evaluating the model’s discriminative capability, offering a comprehensive view of classification performance across various thresholds. Together, these interpretive and evaluative approaches reinforced the model’s credibility and ensured its stability and generalizability.

### Statistical analysis

Continuous variables were summarized as either mean ± standard deviation (Mean ± SD) or median with interquartile range (IQR), depending on their distribution. Group differences were evaluated using independent samples t-tests for normally distributed data or the Mann–Whitney U test for non-normally distributed variables. Categorical variables were expressed as frequencies and percentages [N (%)], with between-group comparisons conducted via the chi-square test. A p-value less than 0.05 was considered indicative of statistical significance.

Feature selection based on the Boruta algorithm was performed using the ‘Boruta’ package (version 4.3.1) in R. Model construction, visualization, and performance evaluation with the random forest algorithm were implemented in Python (version 3.11). Descriptive analyses and data handling were conducted using SPSS software (version 27.0, SPSS Inc., USA).

## Results

A total of 574 children diagnosed with ASD were ultimately included in the analysis, following the exclusion of cases with missing data: 34 lacked CARS scores and 2 had no age information. Among the included participants, 288 were under 4 years of age, while 286 were aged 4 years or older. Random forest models were constructed separately for these two age groups, with ASD severity serving as the target variable. Detailed demographic and clinical characteristics are summarized in Table 1.

**Table 1:** The demographic characteristics of the 574 children with ASD and baseline scores of different age groups

	Total	<4 years old	≥ 4 years old	t	*P*
n(%)	574	288 (50.17)	286 (49.83)		
Age (years) mean ± SD		3.13 ± 0.55	5.25 ± 1.08		
Gender					
Male	463	225 (48.60)	238 (51.40)		
Female	100	57 (57.00)	43 (43.00)		
Gesell developmental scale					
General DQ	60.68 ± 17.35	67.15 ± 15.69	54.16 ± 16.49	9.672	<0.01
Adaptive DQ	61.21 ± 19.57	68.04 ± 17.77	54.31 ± 18.89	8.964	<0.01
Gross motor DQ	70.65 ± 18.17	78.98 ± 16.9	62.24 ± 15.33	12.408	<0.01
Fine motor DQ	68.34 ± 20.22	76.09 ± 19.31	60.51 ± 18.01	9.985	<0.01
Language DQ	46.11 ± 19.43	50.18 ± 18.41	41.99 ± 19.59	5.154	<0.01
Social-behavior DQ	57.61 ± 18.76	62.48 ± 17.75	52.68 ± 18.51	6.473	<0.01
Childhood Autism Rating Scale					
CARS total score	35.23 ± 7.01	35.67 ± 6.54	34.78 ± 7.44	1.522	0.128
Social Responsiveness Scale					
Social awareness	11.63 ± 3.12	11.51 ± 2.98	11.75 ± 3.26	-0.882	0.378
Social cognition	17.97 ± 4.56	17.74 ± 4.27	18.21 ± 4.84	-1.186	0.236
Social communication	33.01 ± 8.96	32.56 ± 8.57	33.48 ± 9.34	-1.182	0.238
Social motivation	15.6 ± 5.31	15.56 ± 5.49	15.65 ± 5.12	-0.190	0.850
Autistic mannerisms	13.55 ± 6.24	12.89 ± 6.46	14.23 ± 5.93	-2.498	0.013
SRS total score	91.75 ± 23.22	90.26 ± 22.81	93.32 ± 23.57	-1.521	0.129
Autism Behavior Checklist					
Sensory	9.06 ± 6.12	9.03 ± 6.1	9.1 ± 6.15	-0.138	0.890
Relating	14.79 ± 8.56	14.74 ± 8.61	14.84 ± 8.52	-0.136	0.891
Body and object use	10.35 ± 8.76	10.03 ± 8.32	10.73 ± 9.26	-0.871	0.384
Language	13.34 ± 7.42	12.76 ± 7.61	14.04 ± 7.14	-1.906	0.057
Social self-help	11.77 ± 5.62	11.67 ± 5.58	11.9 ± 5.67	-0.454	0.650
ABC total score	59.31 ± 29.28	58.22 ± 28.92	60.61 ± 29.71	-0.898	0.370

For feature selection, we utilized the Boruta algorithm with parameters set to “maxRuns = 100” and “pValue = 0.01” to identify the most informative predictors of symptom severity, as visualized in Figure S1. In the plots, the x-axis denotes the evaluated features, while the y-axis reflects their computed importance scores. Shadow attributes, which serve as reference baselines in the selection process, are illustrated using blue boxplots indicating their minimum, mean, and maximum importance values. Features confirmed as important by the algorithm are highlighted in green, whereas those rejected are shown in red. Features with uncertain status—termed tentative—are represented by yellow boxplots, indicating that the algorithm could not conclusively determine their relevance.

Among children with ASD under the age of 4, a total of 33 variables were evaluated, of which 14 were identified as significant, 6 remained in the tentative category, and 13 were excluded by the Boruta algorithm. In practice, age-inappropriate milestones (e.g., E17–E18 and R10–R15) in the younger age group (under 4 years) were consistently classified as rejected or tentative by the algorithm and were thus excluded from the final model. In the group aged 4 years and above, 16 features were deemed significant, 6 remained tentative, and 11 were excluded. The features confirmed as important in each age group are summarized in Table 2.

**Table 2:** The significant features presented by Boruta algorithm

	Group of children with ASD under 4 years old		Group of children with ASD above 4 years old
E5	Expresses demands by language	E8	Uses inhibitory words
R6	Identifies action words	R6	Identifies action words
R4	Recognizes 1 body part	R8	Size concepts
R8	Size concepts	R5	Identifies 1 picture
R5	Identifies 1 picture	E10	Calls partner by name
E10	Calls partner by name	E4	Names 1 object
R3	Finds what adults refer to	E6	Labels 1 picture
E11	Uses pronoun “I”	R4	Recognizes 1 body part
E7	Uses 3- to 4-word sentences	E5	Expresses demands by language
E9	Uses word “mine”	E16	Uses pronoun “he/she”
E4	Names 1 object	R9	Answer 1 question
E12	Uses 8- to 9-word sentences	E3	Uses 2- to 3-word phrase
E15	Narrates things happened 2- to 3-days ago	R10	Names 3 animals
E8	Uses inhibitory words	R7	Identifies 2 colors
		E9	Uses word “mine”
		E2	Says 10–19 words

Figure S2 illustrates the nested cross-validation and grid search procedures used to determine the optimal hyperparameter configurations for the two models. For children under 4 years old, the optimal setting was identified as max_depth = 4 and n_estimators = 140, while for those aged 4 years and above, the best-performing configuration was max_depth = 2 and n_estimators = 90. These parameter combinations yielded the highest classification accuracy in their respective models. In both age groups, the random forest models were further refined by setting class_weight to ‘balanced’ to address potential class imbalance and selecting “entropy” as the splitting criterion. All remaining hyperparameters were retained at their default values.

**Fig. 2:**
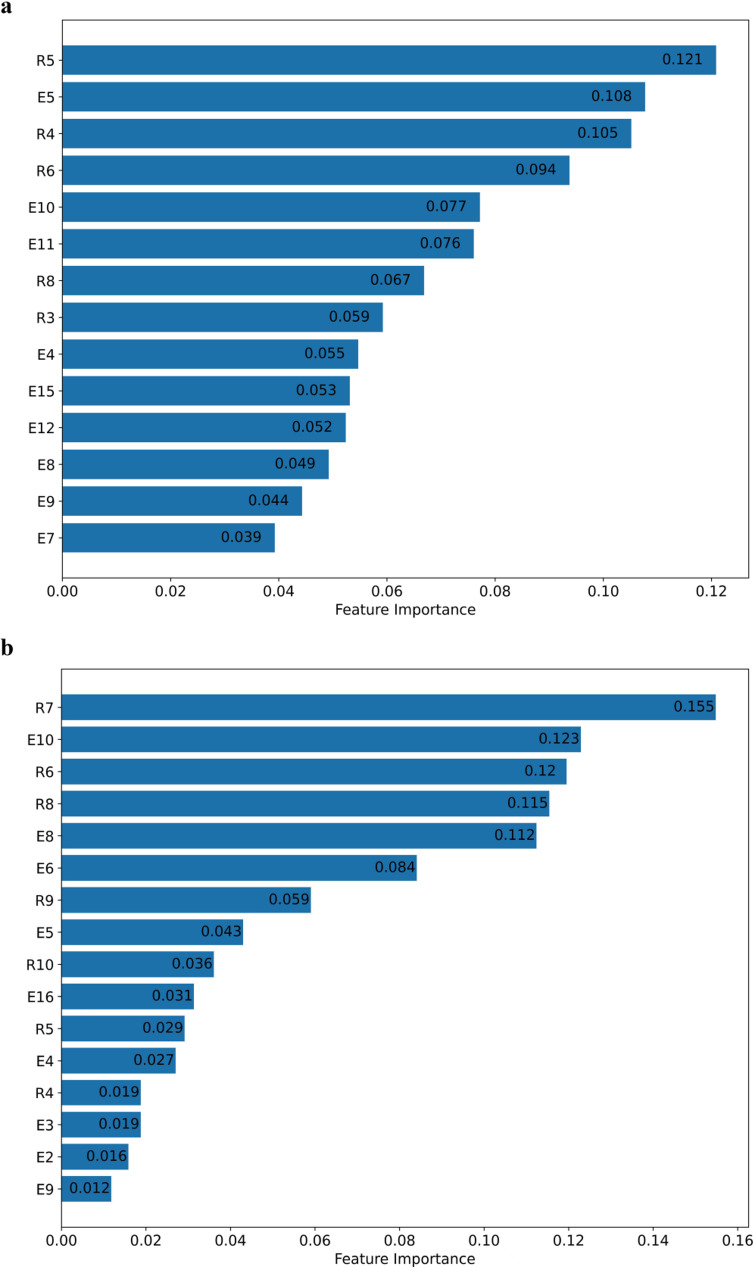
Feature importance ranking for prediction model performance. **a** Group of children with ASD under 4 years old. **b** Group of children with ASD above 4 years old

Upon determining the optimal hyperparameter configuration, random forest models were constructed for each age group. The feature importance plots (Fig. 2) illustrate the relative contribution of each predictor within the respective models. Among children with ASD under the age of 4, the top three influential features were “Identifies 1 picture (R5)”, “Expresses demands by language (E5)”, and “Recognizes 1 body part (R4)”, with corresponding importance scores of approximately 0.121, 0.108, and 0.105, respectively. In contrast, for children aged 4 years and older, the most critical features included “Identifies 2 colors (R7)”, “Calls partner by name (E10)”, “Identifies action words (R6)”, “Size concepts (R8)”, and “Uses inhibitory words (E8)”, with respective importance scores of around 0.155, 0.123, 0.120, 0.115, and 0.112.

The random forest model for the younger age group (under 4 years) consisted of 140 decision trees. Two representative trees were visualized to demonstrate the internal decision structure and logic of the model. For the older age group (4 years and above), the model was composed of 90 decision trees, with selected examples illustrated in Figure S3.

**Fig. 3:**
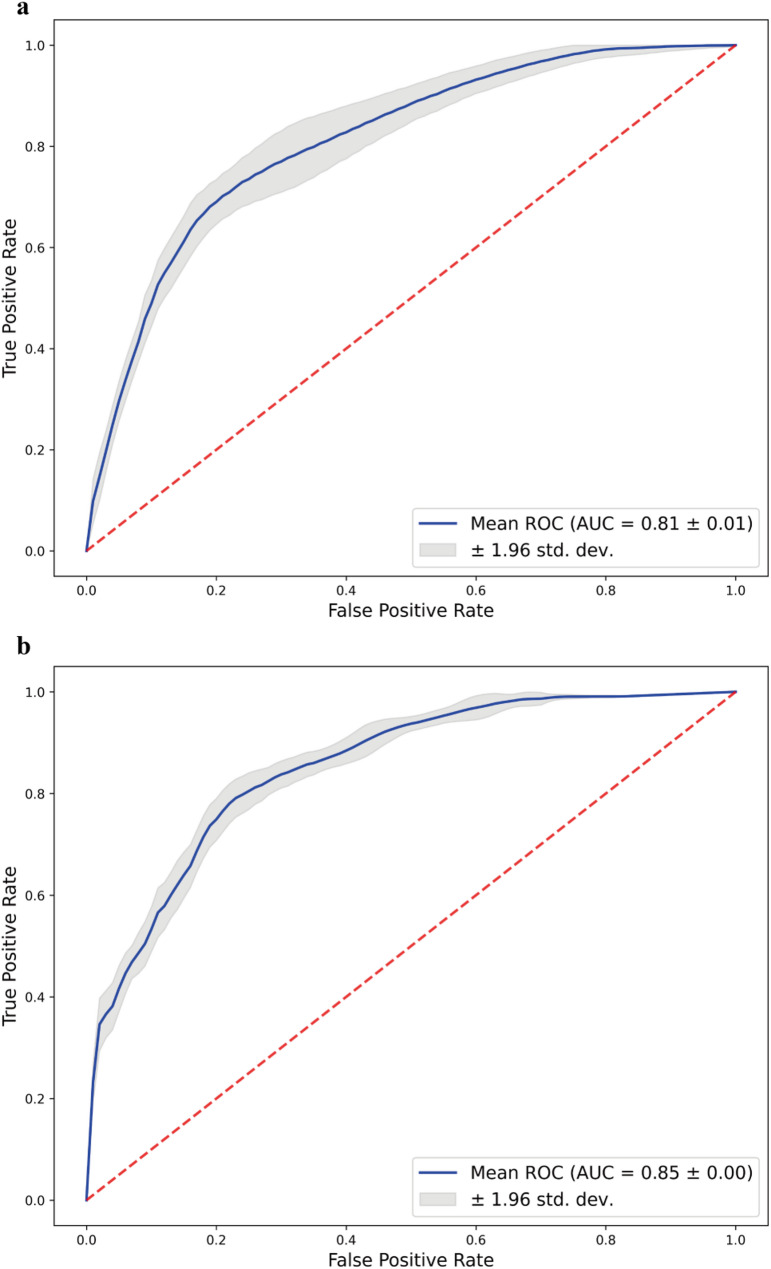
Bootstrap aggregate ROC curves for prediction model performance. (a) Group of children with ASD under 4 years old. (b) Group of children with ASD above 4 years old

Figure 3 presents the ROC curves for the two age-specific models, generated based on 1,000 bootstrap replications to evaluate their discriminative performance. The mean ROC curve, depicted by the blue line, indicates strong classification ability in distinguishing between positive and negative cases. The AUC was 0.81 ± 0.01 for children under 4 years old and 0.85 ± 0.00 for those aged 4 years and above, suggesting high predictive accuracy in both models. The shaded grey region surrounding each ROC curve represents the 95% confidence interval, providing a visual indication of the precision of the AUC estimates. The consistent elevation of the curves above the diagonal red dashed line (representing chance-level performance) further confirms the robust discriminative power of the models. Moreover, the narrow confidence intervals highlight the reliability and stability of the models across different resampled datasets. Additional evaluation metrics—including sensitivity, specificity, precision, and F1 score—along with their corresponding 95% confidence intervals, are summarized in Table S2.

## Discussion

In this study, our random forest analysis demonstrated that the acquisition of specific language milestones can serve as predictors of ASD severity in children. This work highlights the utility of machine learning—particularly the random forest algorithm—in modeling complex, multidimensional, and nonlinear data. By applying this approach, we provide new insights into how language developmental milestones contribute to the stratification of ASD severity. However, it is important to note that our model was constructed within an ASD population and is not intended for diagnostic use across different neurodevelopmental disorders.

The random forest model demonstrates strong capability in identifying key predictive variables of ASD severity in children. Notably, instead of simply treating the attainment of a specific milestone as a binary predictor, we calculated the deviation between the theoretically expected and the actual acquisition status of each language milestone in children with ASD. This approach assigns meaningful values to address whether the absence of a skill is due to the child’s developmental delay or merely age-related limitations. As such, the resulting variables capture not only language delays but also atypical language development patterns [31], while mitigating age-related bias in milestone acquisition.

Nevertheless, we stratified children with ASD into age-based groups during model construction, as longitudinal studies have shown that language ability at age four is the most reliable predictor of later language outcomes in ASD [32], marking a developmental stage where language skills become more stable and discriminative for prognosis. Consistent with this rationale, our findings indicate that the predictive value of specific language milestones for ASD severity differs across the two age-based subgroups.

For children under 4 years of age, the random forest model predicted ASD severity based on the acquisition of 14 language milestones, achieving an AUC of 0.81 ± 0.01. Three variables demonstrated feature importance greater than 10%, namely: “Identifies 1 picture”, “Expresses demands by language”, and “Recognizes 1 body part”. These language milestones are closely associated with pragmatic language development. While “Identifies 1 picture” and “Recognizes 1 body part” primarily reflect receptive language abilities, their successful completion also requires broader social-communicative processes. To identify a picture or a body part when prompted, a child must not only comprehend the verbal label but also establish a shared attentional frame with the examiner, shifting gaze or orientation toward the relevant referent. In many clinical contexts, children respond by pointing to the correct picture or by touching the named body part, behaviors that inherently involve gesture use and coordinated attention. Research has shown that receptive language delays are often more pronounced in children with ASD and may reflect a characteristic developmental pattern in this population [33, 34]. Declarative pointing is a key component of joint attention and represents a core ability in social sharing and interaction [35, 36]. Pointing to objects has been shown to significantly predict children’s future vocabulary development [37]. Young children initially use gestures to express referents, and these gesture-based referents are typically transformed into spoken words [38]. “Expresses demands by language” serves as a critical pragmatic language milestone. Pragmatic language impairments are currently recognized as a core linguistic feature of the ASD [39, 40], and its absence is associated with higher symptom severity in ASD [41]. Therefore, the identification of these language milestones as key predictors of ASD severity in children under the age of four may provide useful insights for guiding interventions and informing developmental support strategies.

In children aged 4 years and above, the random forest model predicting ASD severity based on the acquisition of 16 specific language milestones achieved an AUC of 0.85 ± 0.00, outperforming the model for children under 4. This suggests that as language development becomes more stable, language milestones serve as stronger predictors of ASD severity. Five variables demonstrated feature importance exceeding 10%. The most influential language milestones included “Identifies 2 colors”, “Calls partner by name”, and “Identifies action words”. Consistent with the findings in the younger age group, these milestones are primarily related to receptive language, nonverbal communication, and pragmatic language abilities in children with ASD. Notably, among expressive language milestones, “Calls partner by name” emerged as the strongest predictor of ASD severity. The acquisition of this milestone reflects pragmatic language development, encompassing pronoun use and referential expression. Research consistently shows that individuals with ASD frequently avoid or misuse personal names and pronouns [42], often exhibiting pronoun reversal [43]. This pattern persists throughout childhood and adolescence and is specifically associated with social-pragmatic deficits rather than general language delay [44]. Among other items on pronoun and referential expression use (E11, E13, E16), only “Uses pronoun he/she” (E16) was retained in this cohort, reflecting that third-person pronouns require more advanced pragmatic skills and thus continue to differentiate ASD severity at later stages. Moreover, the predictive model reinforces our earlier hypothesis that the predictive power of language in ASD lies primarily in pragmatic rather than structural language abilities. Although our expressive language domain included milestones such as “Says 1 word” “Uses 2- to 3-word phrase” and “Uses 8- to 9-word sentences”, these structural language milestones were incorporated into the model but did not emerge as the most influential predictors. Thus, the predictive value of these pragmatic milestones suggests their potential relevance for assessing symptom severity and informing intervention planning in older children with ASD.

If early language milestones can meaningfully predict the ASD severity, they may provide valuable insights for both research and clinical practice. Standardized diagnostic tools such as the CARS, ADI-R and ADOS are widely used to assess severity, but these evaluations often occur later and require considerable time, expertise, and resources. As a result, especially in resource-limited settings or under long wait times, timely assessment of ASD severity is frequently delayed [45]. Existing early screening tools primarily identify ASD risk but rarely provide information on symptom severity [46], creating a gap in early clinical decision-making, since severity directly influences intervention intensity, planning, and prognosis. To address this, our study proposes a predictive model of ASD severity based on early language milestones, aiming to extend the utility of early severity stratification and to provide an additional reference for individualized intervention planning. In practice, predicted severity could help clinicians calibrate support levels—for example, children with milder severity may benefit from parent-mediated interventions, whereas those with greater severity may require higher-intensity programs and multidisciplinary input.

This study has several limitations. First, it was a cross-sectional analysis, so causal inferences cannot be drawn and the stability of the predictive model over time remains unknown. Longitudinal studies are needed to validate its performance in follow-up assessments and to examine long-term developmental outcomes. Second, although our findings likely generalize across languages, cross-linguistic variability may influence the timing or expression of these skills; future studies should validate the model in diverse linguistic and cultural contexts. Third, transforming milestone discrepancies into binary variables may lead to a loss of information on the degree of delay or advancement. Future studies may benefit from modeling these differences as continuous variables, which could provide more detailed insights. Fourth, the predictors were limited to language milestones derived from the GDS and may not fully capture pragmatic, gestural, or broader socio-communicative behaviors. Including a broader range of such measures in future studies may improve model accuracy. Fifth, we did not conduct stratified analyses by gender. Our sample reflected the typical male-to-female prevalence ratio in ASD (4.6:1), leaving too few girls for meaningful stratified analyses. Finally, although relatively large for an ASD clinical study, our sample remains modest from a machine learning perspective. Models with multiple features and subgroup stratifications require larger and more diverse datasets to improve generalizability and reduce overfitting.

In conclusion, this study is the first to apply a machine learning algorithm to predict ASD severity in children based on the acquisition of specific early language milestones. This approach may facilitate early estimation of ASD severity and provide an additional reference for individualized intervention planning, particularly in settings where access to comprehensive severity assessments is limited.

## Supplementary Information

Below is the link to the electronic supplementary material.

Supplementary Material 1

## Data Availability

No datasets were generated or analysed during the current study.
