# Supplementary material for: Predicting autism spectrum disorder severity in children based on specific language milestones: a random forest model approach
Source: Child Adolesc Psychiatry Ment Health. 2025 Nov 18;19:127. doi: 10.1186/s13034-025-00988-0 (PMC12625304; doi:10.1186/s13034-025-00988-0)
Supplement: Supplementary file 1 — Supplementary Material 1 [file 13034_2025_988_MOESM1_ESM.docx]

**Supplementary Figures**


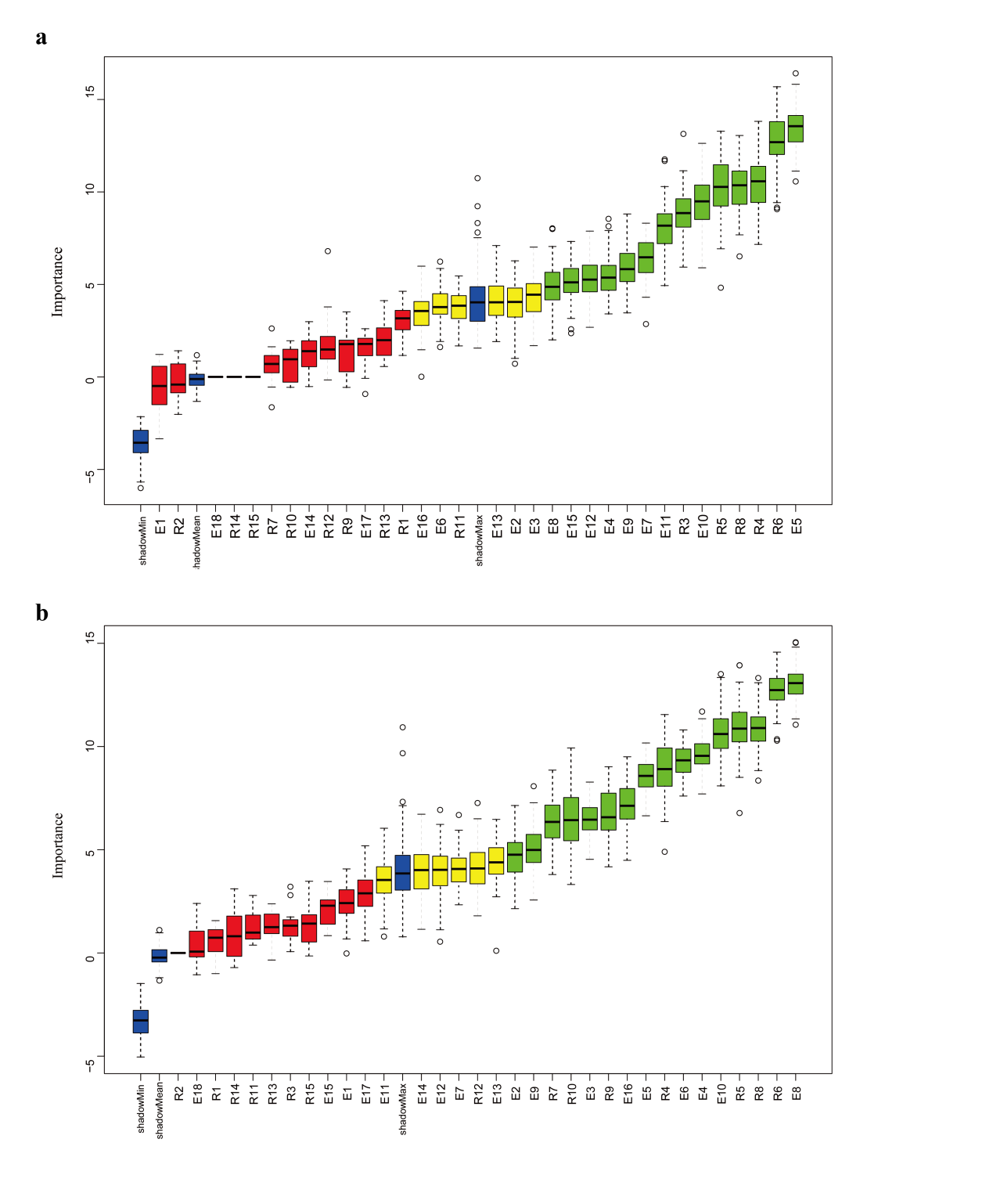


**Figures S1.** Feature selection using the boruta algorithm. (a) Group of children with ASD under 4 years old. (b) Group of children with ASD above 4 years old.


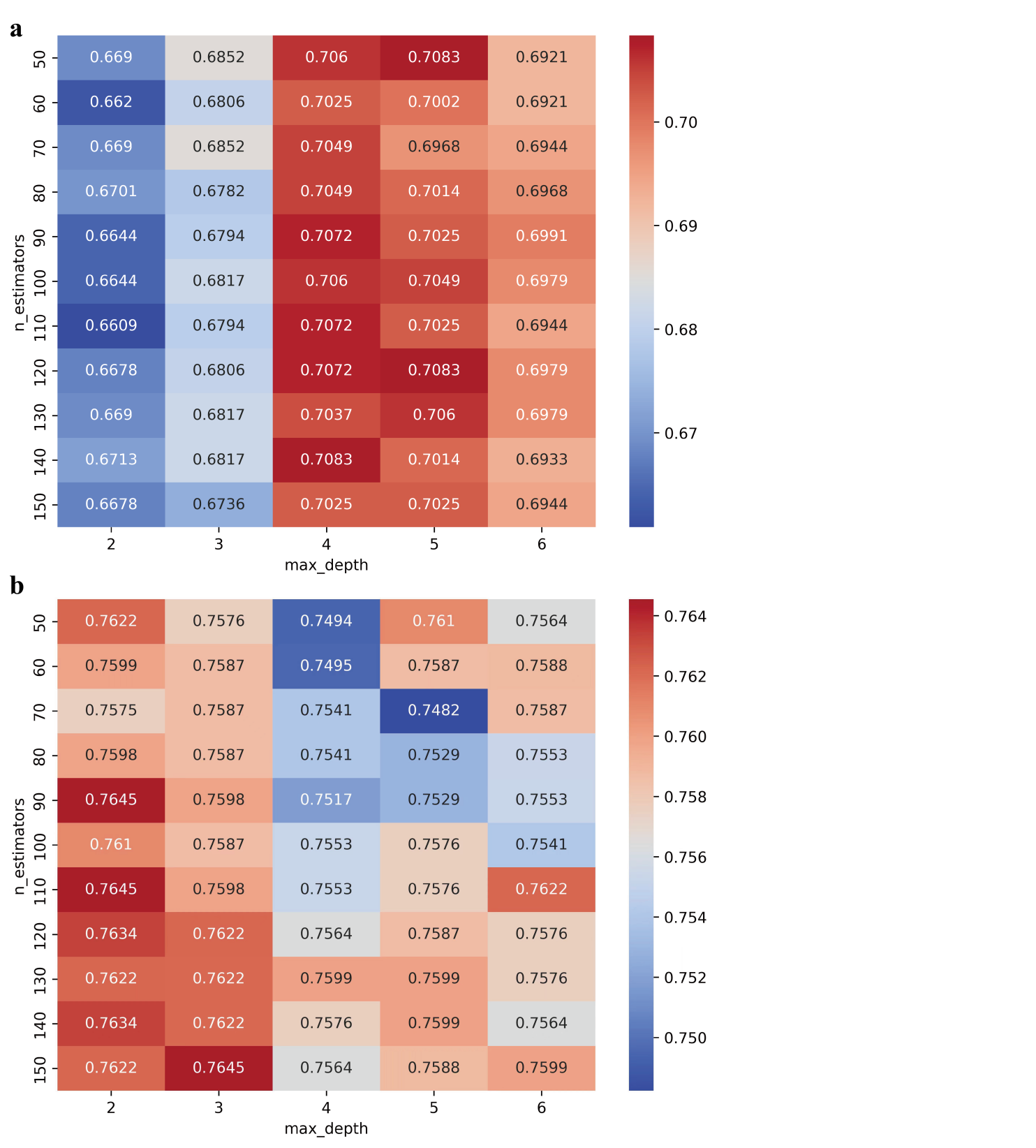


**Figure S2.** Optimal hyperparameter tuning of random forest model: influence of “max_depth” and “n_estimators” on model accuracy. The horizontal and vertical axes represent the depth of the trees and the number of trees in the random forest model, respectively. (a) Group of children with ASD under 4 years old. (b) Group of children with ASD above 4 years old.

**
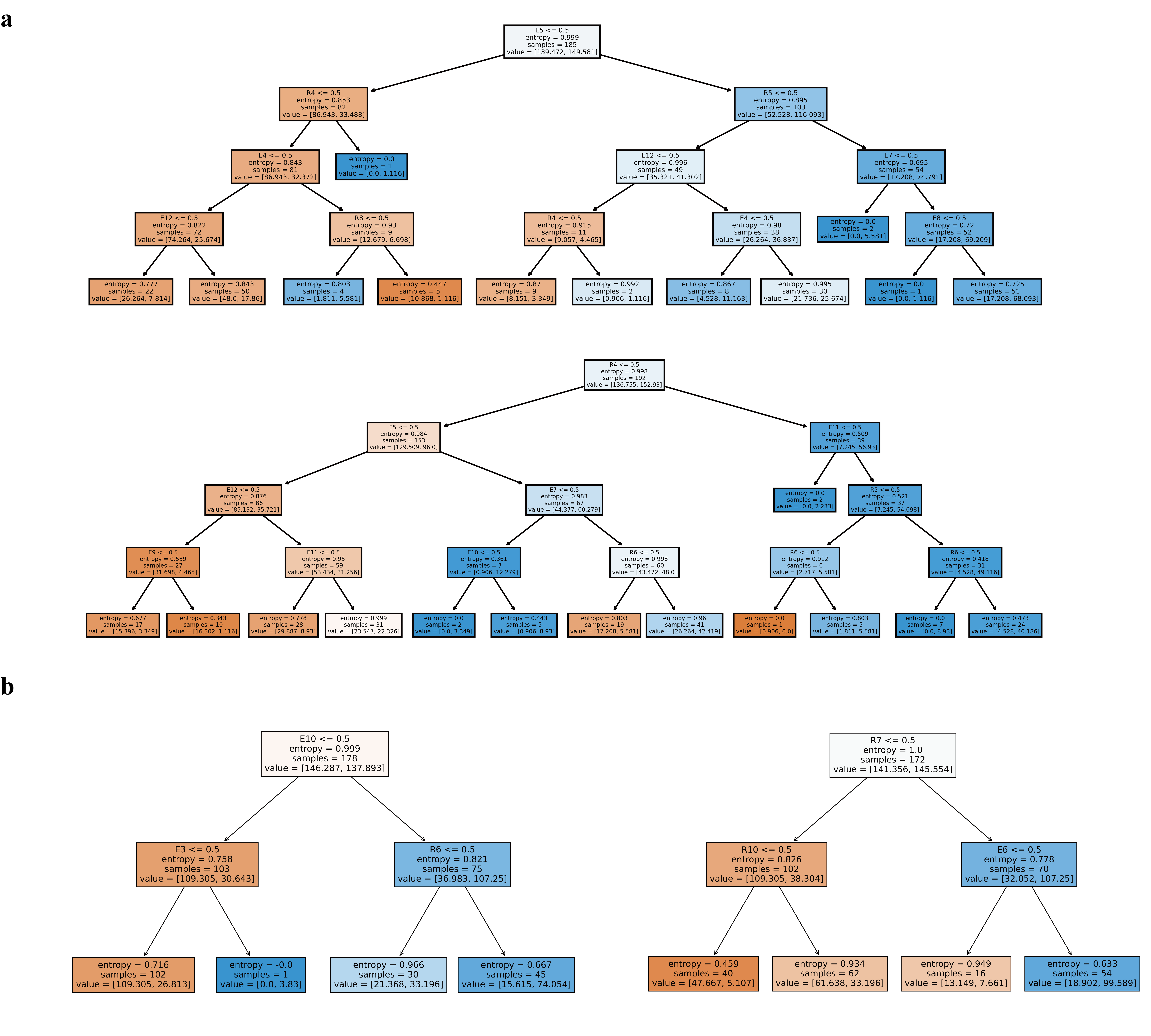
Figure S3.** Visualization of two trees from each random forest composed. (a) Group of children with ASD under 4 years old. (b) Group of children with ASD above 4 years old.

**Table S1.** The identified expressive and receptive language milestones of children with ASD aged 2-7 years old by Mokken scale analysis.

|  | Expressive Language Items | Standard acquisition age |  | Receptive Language Items | Standard acquisition age |
| --- | --- | --- | --- | --- | --- |
| E1 | Says 1 word | 36week | R1 | Waves goodbye | - |
| E2 | Says 10-19 words | 15month | R2 | Gives toy on verbal request | - |
| E3 | Uses 2- to 3-word phrase | 18month | R3 | Finds what adults refer to | 48week |
| E4 | Names 1 object | 18month | R4 | Recognizes 1 body part | 52week |
| E5 | Expresses demands by language | 18month | R5 | Identifies 1 picture | 15month |
| E6 | Labels 1 picture | 21month | R6 | Identifies action words | 30month |
| E7 | Uses 3- to 4-word sentences | 21month | R7 | Identifies 2 colors | 36month |
| E8 | Uses inhibitory words | 21month | R8 | Size concepts | 30month |
| E9 | Uses word “mine” | 21month | R9 | Answer 1 question | 36month |
| E10 | Calls partner by name | 18month | R10 | Names 3 animals | 48month |
| E11 | Uses pronoun “I” | 24month | R11 | Identifies 6 object functions | 48month |
| E12 | Uses 8- to 9-word sentences | 30month | R12 | Comprehends 3 antonyms | 42month |
| E13 | Uses pronoun “you" | 24month | R13 | Comprehends 6 verbs | 42month |
| E14 | Uses “and/but” connect sentences | 36month | R14 | General knowledge (2/3) | 60month |
| E15 | Narrates things happened 2- to 3-days ago | 30month | R15 | Comprehends 4 nouns (4/6) | 60month |
| E16 | Uses pronoun “he/she” | 30month |  |  |  |
| E17 | Repeats 13-word sentences | 48month |  |  |  |
| E18 | Narrates according to the picture | 72month |  |  |  |

**Table S2.** Performance metrics for prediction model with 95% confidence intervals.

|  | Group of children with ASD under  4 years old, Value (95% CI) | | Group of children with ASD above  4 years old, Value (95% CI) |
| --- | --- | --- | --- |
| AUC | 0.812 (0.794 - 0.828) | 0.853 (0.848 - 0.857) | |
| Sensitivity | 0.721 (0.651 - 0.798) | 0.715 (0.634 - 0.786) | |
| Specificity | 0.760 (0.642 - 0.830) | 0.816 (0.759- 0.856) | |
| Precision | 0.712 (0.636 - 0.760) | 0.716 (0.674 - 0.745) | |
| F1 Score | 0.715 (0.679 - 0.748) | 0.715 (0.679 - 0.744) | |
